# Bisphosphonates Prescription for Patients With Hip Fractures Based on Evaluation by a Dentist

**DOI:** 10.7759/cureus.35407

**Published:** 2023-02-24

**Authors:** Hayato Inoue, Ryunosuke Oyama, Kimitaka Nakamura, Akihiko Inokuchi, Takahiro Hamada, Teiyu Izumi, Ryuta Imamura, Toshihiro Ebihara, Takahiro Inoue, Takeshi Arizono

**Affiliations:** 1 Department of Orthopedic Surgery, Kyushu Central Hospital of the Mutual Aid Association of Public School Teachers, Fukuoka, JPN

**Keywords:** clinical pathway, osteonecrosis of the jaw, dentist, hip fractures, bisphosphonates

## Abstract

Background

The purpose of this study is to clarify the current status of the prescription of postoperative bisphosphonates for patients with hip fractures and to explore the factors that prevent the postoperative prescription of bisphosphonates.

Methods

Of 180 patients with hip fractures treated surgically at our hospital between August 2019 and April 2020, 149 patients (46 men and 103 women; mean age: 83.9 ± 9.0 years), excluding 31 patients already prescribed bisphosphonates or denosumab, were included in the study. All patients were treated based on our clinical pathway, and their risk of jaw osteonecrosis was evaluated prior to the initiation of bisphosphonates by a dentist in our hospital. We collected data from the medical records on osteoporosis treatment interventions at admission and discharge, the reasons why postoperative bisphosphonates could not be prescribed at discharge, the proportion of patients who had follow-ups at our hospital, and patients’ osteoporosis treatment status.

Results

Eighteen (12.8%) and 95 (63.8%) patients were prescribed anti-osteoporosis drugs at admission and discharge, respectively. One hundred and twenty-one patients (86.8%) could not be prescribed postoperative bisphosphonates at discharge - 71 (58.7%) because of oral hygiene problems, 34 (28.1%) because they did not have regular dental consultations, seven (5.8%) because of renal dysfunction, eight (6.6%) because of poor cognitive and swallowing function, and one (0.8%) because of medication side effects. Forty-nine patients (32.9%) went to our hospital for follow-up and 11 were introduced to bisphosphonates or denosumab at follow-up.

Conclusions

The number of patients with hip fractures who were prescribed postoperative bisphosphonates was low in our study. The oral hygiene problems identified by dentists accounted for responsible for the low prescription rate of postoperative bisphosphonates. Therefore, coordination with dentists may be important to increase the postoperative bisphosphonate prescription.

## Introduction

The number of fragility fractures based on osteoporosis is increasing year by year with the rapid aging of the population in Japan. The annual number of hip fractures in Japan is estimated to be about 250,000 in 2020 and about 300,000 in 2030 [1]. Most hip fractures in the elderly are fragile fractures based on osteoporosis, so it goes without saying that it is important for patients who were once diagnosed with fragility fractures to receive treatment for osteoporosis to prevent secondary fractures. The prevention and treatment guidelines for osteoporosis treatment in Japan recommend bisphosphonates as first-line agents for patients with hip fractures [2,3].

In recent years, the importance of postoperative intervention with anti-osteoporosis drugs for patients with fragility fractures has been emphasized, and the approaches through multidisciplinary collaborations such as liaison services have been reported to be effective medically and financially [4]. However, the efforts vary by medical area or hospital, so patients with fragility fractures have not received sufficient treatment for osteoporosis [5-7]. It seems that there are various complicated problems for inadequate interventions, such as the follow-up for treatment, side effects of bisphosphonates, diagnosis procedure combination (DPC), the lack of interest in osteoporosis, and so on. The present study was a descriptive study with data collected retrospectively. The purpose of this study is to clarify the current status of the prescription of postoperative bisphosphonates for patients with hip fractures and to explore the factors that prevent the postoperative prescription of bisphosphonates.

## Materials and methods

We retrospectively reviewed the data of 180 consecutive patients who were treated surgically in our clinic from August 2019 to April 2020. Among them, we excluded 31 patients with a preoperative prescription of bisphosphonates or denosumab. A total of 149 patients (46 men and 103 women) were finally enrolled in the present study. The mean age of the study population was 83.9 ± 9.0 years.

Fifty-six cases were trochanteric fractures (including subtrochanteric fractures), and 93 cases were cervical fractures (including cervical base fractures). The mean YAM% (young adult mean) scores for dual X-ray absorptiometry (DXA) scans performed after the surgery were 59.3 (12.6%) for the femur and 78.5 (17.9%) for the lumbar.

All patients were treated based on our clinical pathway and their risk of osteonecrosis of the jaw prior to the introduction of bisphosphonates was evaluated by a dentist at our hospital. If it was decided that they were relatively safe, oral bisphosphonates were prescribed in the hospital. They were transferred to convalescent hospitals. When they were discharged from our hospital, the doctors made the reservations for follow-up or told them (or their families) to go to our hospital for follow-up after discharge from convalescent hospitals.

We collected data from medical records, including the osteoporosis treatment intervention at admission and discharge, the reasons why postoperative bisphosphonates could not be prescribed at discharge, the proportion of patients who went to our hospital for follow-up, and their status of treatment for osteoporosis.

This study was approved by the Kyushu Central Hospital Review Board on Clinical Research Plans (approval number 242) and conducted at Kyushu Central Hospital, Fukuoka, Japan.

## Results

**Figure 1:**
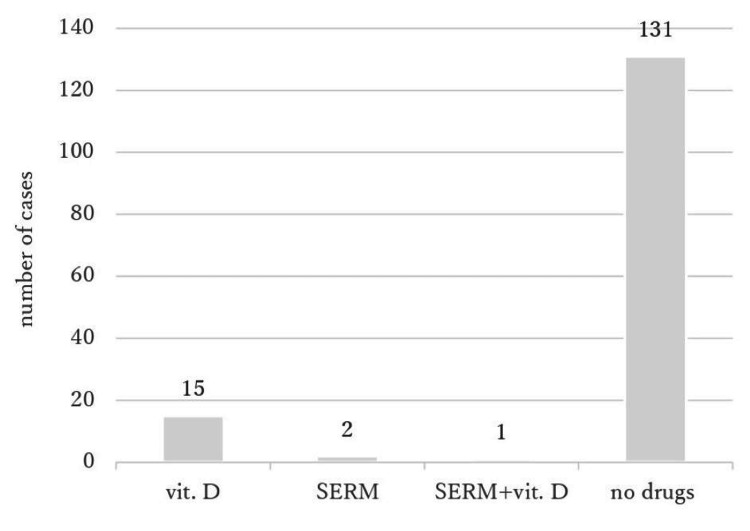
Prescribed anti-osteoporosis drugs at admission vit.D: vitamin D, SERM: selective estrogen receptor modulator

**Figure 2:**
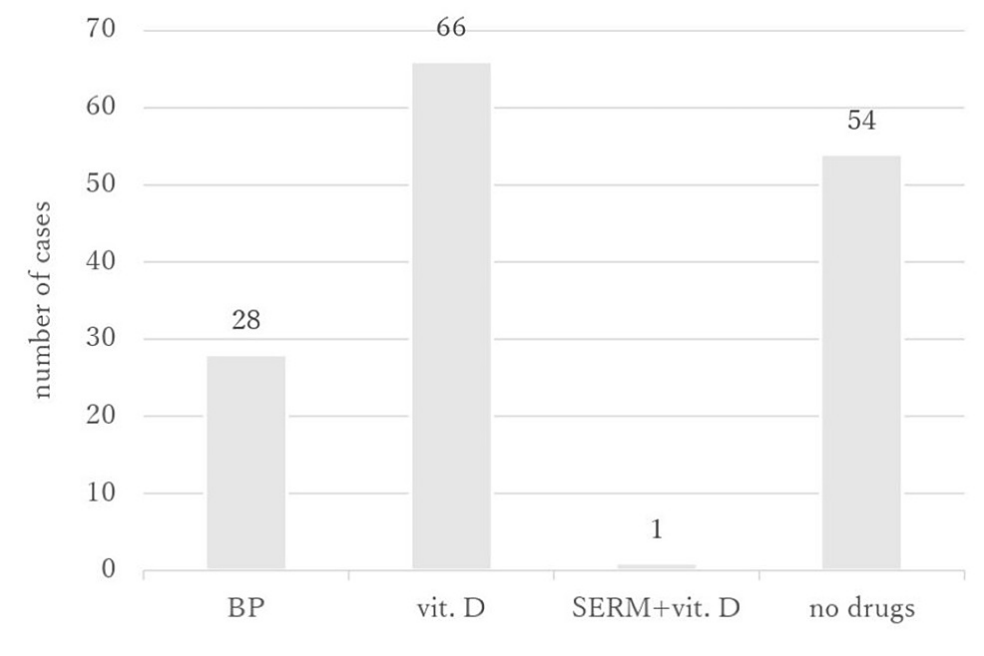
Prescribed anti-osteoporosis drugs at discharge BP: bisphosphonate, vit.D: vitamin D, SERM: selective estrogen receptor modulator

Eighteen patients (12.8%) were prescribed anti-osteoporosis drugs at admission. Fifteen received active vitamin D3, two received selective estrogen receptor modulators, and one received both (Figure 1). Ninety-five patients (63.8%) were prescribed anti-osteoporosis drugs at discharge. Twenty-eight (18.8%) received oral bisphosphonates, 66 received active vitamin D3, and one received both selective estrogen receptor modulators and active vitamin D3 (Figure 2).

**Figure 3:**
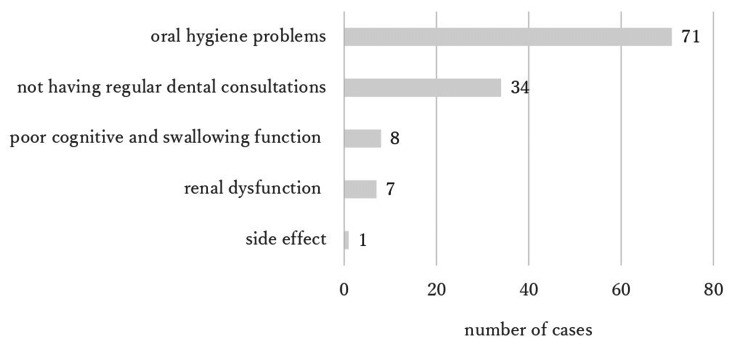
The reasons why postoperative bisphosphonates could not be prescribed at discharge

One hundred and twenty-one patients (86.8%) could not be prescribed postoperative bisphosphonates at discharge. This was because of oral hygiene problems in 71 patients (58.7%), a lack of regular dental consultations in 34 (28.1%), poor cognitive and swallowing function in eight (6.6%), renal dysfunction in seven (5.8%), and medication side effects in one (0.8%) (Figure 3).

**Figure 4:**
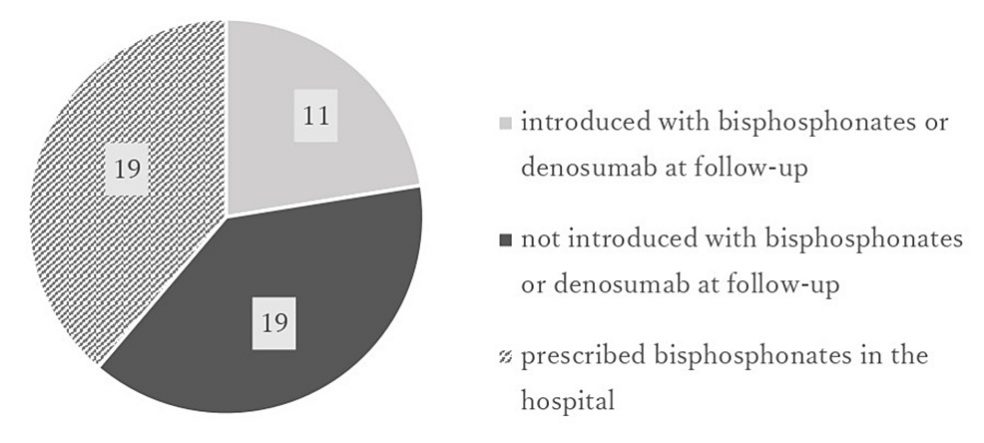
Number of patients who went to our hospital for follow-up (49 total patients)

Forty-nine patients (32.9%) went to our hospital for follow-up after discharge from convalescent hospitals. Of these, 30 patients were not prescribed postoperative bisphosphonates and 11 were taking bisphosphonates or denosumab at follow-up (Figure 4).

## Discussion

In this study, we made two important observations. First, the number of patients with hip fractures who were prescribed postoperative bisphosphonates was low (18.8%), and most patients could not be prescribed postoperative bisphosphonates at discharge because of oral hygiene problems or lack of regular dental consultations. Second, more than half of the patients who were treated surgically at acute care hospitals did not go to the hospital for follow-up after discharge from convalescent hospitals.

For hip fractures, approaches to prevent secondary fractures, such as contralateral fractures or vertebral fractures, are extremely important, and the treatment for osteoporosis should be initiated as soon as possible. Various reports have discussed the incidence of contralateral fractures, and recent studies show that the incidence is around 5% [8-10]. Once patients have contralateral fractures, there is a high risk of being bedridden or dying because of a severe drop in activities of daily living [11,12].

Bisphosphonates such as alendronate and risedronate should be given to patients with hip fractures as a first-line agent based on their effectiveness in the prevention of secondary hip fractures [2,3]. However, one potentially serious side effect of bisphosphonates is osteonecrosis of the jaw, which is characterized by refractory bone exposure [13]. Therefore, in 2019, we began a protocol in which all patients with hip fractures were examined by a dentist to evaluate their oral hygiene and risk before initiating postoperative bisphosphonates.

As a result, in many cases, bisphosphonates were deemed unsafe because of problems with oral hygiene (71 patients (58.7%)) or dental consultation status (34 patients (28.1%)). Most patients with hip fractures are elderly and their oral self-care seems to be diminished, likely because of deterioration in physical function. Thus, many patients have issues with tooth decay or periodontitis.

Osteonecrosis of the jaw related to bisphosphonates was first reported in 2003 [14], and various other studies have confirmed the results. Khan et al. conducted a systematic review of the literature on anti-resorptive agent-related osteonecrosis of the jaw that occurred from 2003 to 2014 [15]. In this study, they reported that the incidence of osteonecrosis of the jaw associated with bisphosphonates was 1.04-69 per 100,000, and with denosumab, it was 0-30.2 per 100,000. They concluded that the incidence of osteonecrosis of the jaw in osteoporosis patients was low, and only slightly higher than in the general population.

It is not possible to simply compare the risk of secondary fractures with the risk of jaw osteonecrosis associated with bisphosphonates, but if the oral conditions are age-appropriate, patients may benefit from introducing bisphosphonates for the treatment of osteoporosis as soon as possible postoperatively. If immediate oral intervention is needed, this treatment should be prioritized before the introduction of bisphosphonates, and regular oral care is required thereafter [13]. However, because of concerns about the risk of osteonecrosis of the jaw, there were many patients in whom postoperative bisphosphonates could not be prescribed after surgery, and they thus remained untreated for osteoporosis.

Based on our results, the percentage of patients who are in a state in which it is difficult to introduce bisphosphonates is high based on an oral evaluation by a dentist, and it may be hard in cases in which appropriate oral care after discharge cannot be expected. In particular, when they are discharged to a geriatric health services facility or special nursing home for the elderly, it may be difficult to obtain a dental examination for oral care purposes. While dentists are sensitive to the assessment of osteonecrosis of the jaw, they may not be aware of the need for osteoporosis treatment. Mutual understanding and close cooperation between orthopedic surgeons and dentists are important for risk management before the introduction of bisphosphonates and for subsequent oral care. We considered asking family doctors about both the introduction of bisphosphonates and regular oral care by dentists to increase the rate of postoperative bisphosphonate use.

There are other problems associated with the treatment of osteoporosis after hip fracture. In many cases, neither orthopedic surgeons nor family doctors are able to follow up closely with patients after discharge from an acute care hospital regardless of the presence or absence of postoperative osteoporosis treatment. In this study, only 49 out of 149 patients (32.9%) followed up at our hospital, and we do not know if these patients have been receiving treatment for osteoporosis. Even in cases in which bisphosphonates were introduced in the hospital, it is quite possible that patients did not continue them after discharge. Ganboa et al. reported that about 65% of patients who were prescribed oral bisphosphonates in acute care hospitals after a hip fracture continued this treatment one year later. This remains a major issue if patients continue their postoperative bisphosphonate treatment after initiation in the acute care hospital setting.

The more serious problem is that of patients who could not be prescribed oral bisphosphonates in acute care hospitals and did not follow up at our hospital. What medical institution would initiate treatment for osteoporosis after discharge? If orthopedic surgeons coordinate well with family doctors, they will take care of the treatment, but this is difficult with the current system at our hospital. It appears that there are a large number of patients who remain untreated for osteoporosis after surgery.

As described above, how to intervene and continue treatment for osteoporosis after discharge from acute care hospitals is an important issue in the treatment of patients with hip fractures. In recent years, multidisciplinary collaborations, such as liaison services, have attracted attention in the treatment of osteoporosis and have been reported to be effective medically and financially [3]. Shimodan et al. conducted a multicenter study on the treatment of osteoporosis after hip fracture during the 10 years from 2008 to 2017 [5]. In this study, with the introduction of an osteoporosis liaison service in 2012, the proportion of those who had outpatient visits after discharge, which had been about 25% until then, gradually increased and reached about 55% in 2017. The rate of introduction of bisphosphonates and/or denosumab also significantly increased over this time period.

The liaison service has also been reported to be cost-effective [16,17], and it is hoped that it will spread to many medical areas in Japan, where a super-aging society is approaching, from the perspective of medical and long-term care costs. We believe it is necessary for these approaches to be carried out with the sharing of information about the treatment of osteoporosis not only within one acute care hospital but also between other facilities such as convalescent hospitals and family doctors. A network should be established to improve the rate of patients who receive and continue treatment for osteoporosis after a fragility fracture is diagnosed.

This study had a limitation. The results of this study are only those of our original approach made in a small acute care hospital, and it is only a descriptive study. These results are not representative of the current status of treatment for osteoporosis in acute care hospitals in Japan. In particular, because our hospital performed a facility-specific effort to assess the risk of jaw osteonecrosis in all patients with hip fractures before introducing postoperative bisphosphonates, our hospital is not of use as a reference. Also, the evaluation of the dental aspect might be inadequate, as it was conducted subjectively by only one dentist.

## Conclusions

The number of patients with hip fractures who were prescribed postoperative bisphosphonates was low in our study. In many patients, postoperative bisphosphonates were not prescribed because of oral hygiene problems identified by the dentist. Coordination with dentists may be important to increase the postoperative bisphosphonate prescription.
